# The DiaActive study: feasibility, safety and acceptability of a fall-preventive rhythm- and ADL-based exercise protocol for older adults with type 2 diabetes

**DOI:** 10.1007/s40520-026-03328-0

**Published:** 2026-02-04

**Authors:** Aksayan Arunanthy Mahalingasivam, Asger Ahlmann Bech, Peter Vestergaard, Martin Grønbech Jørgensen, Nicklas H. Rasmussen

**Affiliations:** 1 Steno Diabetes Center North Denmark, Aalborg University Hospital, Aalborg, Denmark; 2 Department of Clinical Medicine, Aalborg University, Aalborg, Denmark; 3 Department of Geriatric Medicine, Aalborg University Hospital, Aalborg, Denmark

**Keywords:** Type 2 diabetes, Rhythm-based training, Activities of daily living, Feasibility study, Fall prevention

## Abstract

**Purpose:**

Older adults with type 2 diabetes (T2D) face an elevated risk of falls due to combined physical and cognitive impairments. The DiaActive feasibility study evaluated the safety, feasibility, and acceptability of a novel fall-prevention exercise program integrating multitask rhythm-based movement, activities of daily living (ADL) exercises, and a structured social component.

**Methods:**

Eight community-dwelling adults (≥ 65 years) with T2D participated in two 60-minute physiotherapist-led sessions per week for four weeks (total 480 min). The program combined rhythm-based (Rythma) and ADL exercises with structured social interaction. Feasibility outcomes included adherence, safety, satisfaction, and overall acceptability.

**Results:**

Adherence was high, with a median attendance of 88% (range 75–100%) and 100% questionnaire completion. No injuries or adverse events occurred, indicating good safety and tolerability. Participants reported that session difficulty progressed appropriately, shifting from “too easy” early on to “appropriately challenging” by week four. Satisfaction increased over time, with most rating sessions as “satisfactory” or “very satisfactory.” Qualitative feedback evolved from exercise-focused comments to broader reflections on body awareness and social connectedness. The positive group atmosphere and structured social elements were key motivators supporting adherence and engagement.

**Conclusion:**

The DiaActive protocol—combining rhythm-based, ADL-focused, and social components—was safe, feasible, and well accepted by older adults with T2D. High adherence, absence of adverse events, and increasing satisfaction support progression to a fully powered randomized controlled trial to assess clinical efficacy and long-term adherence.

**Supplementary Information:**

The online version contains supplementary material available at 10.1007/s40520-026-03328-0.

## Introduction

### Background

 Diabetes mellitus is an increasingly prevalent disease worldwide and poses a growing burden on healthcare systems [1]. Among its many complications, an elevated risk of falls has emerged as a major concern [2]. This risk is multifactorial and reflects the accumulation of comorbidities and functional impairments that develop with disease progression [1]. Falls and their consequences are important yet often underrecognized contributors to morbidity, mortality, and reduced quality of life in this population.

### Falls and underlying mechanisms

Diabetes-related comorbidities negatively affect postural control in both static and dynamic conditions, with alterations in the center of pressure area identified as a key factor [3]. Impaired postural stability is associated with long disease duration, poor glycaemic control, and dysfunction in the sensory systems essential for balance—vision, proprioception, and the vestibular apparatus [4]. Such dysfunctions are frequently linked to diabetic neuropathy, the most common complication of diabetes [5], which can also cause autonomic dysfunctions such as impaired blood pressure regulation and arrhythmias [6–8]. Additionally, people with diabetes have a higher prevalence of cardiovascular comorbidities, including heart failure, ischemic heart disease, and dyslipidaemia [6]. These conditions share overlapping metabolic and vascular mechanisms with diabetes [9], collectively increasing instability and fall risk [10].

### Fractures and consequences

The increased incidence of falls in diabetes is accompanied by a higher prevalence of fractures compared with people without the disease [11]. Both type 1 (T1D) and type 2 diabetes (T2D) contribute to skeletal fragility through distinct mechanisms, including impaired bone turnover and microarchitectural changes [12]. Consequently, the combination of frequent falls and reduced bone strength results in greater fracture risk, higher mortality, and diminished quality of life [13].

### Preventive strategies and study rationale

Many diabetes-related comorbidities are modifiable, and physical exercise remains one of the most effective interventions for improving metabolic health and preventing complications [14]. Beyond these systemic effects, exercise reduces fall incidence by enhancing postural control and functional capacity [15, 16]. When combined with cognitive or executive tasks—such as dual-task or rhythm-based training—benefits extend to improved coordination and cognitive–motor integration [17–20]. Rhythm-based programs like Dalcroze Eurhythmics have shown fall-preventive benefits in older adults [21–23], and when delivered in socially engaging group settings, they can improve motivation and adherence [24].

### Aim of this project

The primary aim of this study was therefore to evaluate the feasibility, safety, and acceptability of the DiaActive fall-preventive exercise protocol for older adults with T2D as preparation for a future randomized controlled trial. This collaborative process is intended to refine both the exercise and social components, ultimately optimizing the intervention program and finalizing an exercise protocol for the upcoming randomized controlled trial.

## Outcomes

Primary feasibility outcomes were predefined as (1) ≥ 75% session attendance, (2) absence of serious adverse events, and (3) high participant-rated acceptability.

## Methods

### Study design

This single-arm feasibility study was conducted at Steno Diabetes Center North Denmark (SDCN) between 19 May and 17 June 2025 to evaluate the safety, acceptability, and practical feasibility of the DiaActive fall-preventive exercise protocol prior to a randomized controlled trial. The study was designed as a process- and acceptability-oriented investigation, not to assess intervention effects. The study was conducted among adults aged ≥ 65 years with T2D. All sessions took place at SDCN and were supervised by trained physiotherapists experienced in exercise protocol for older adults.

### Intervention

**Fig. 1:**
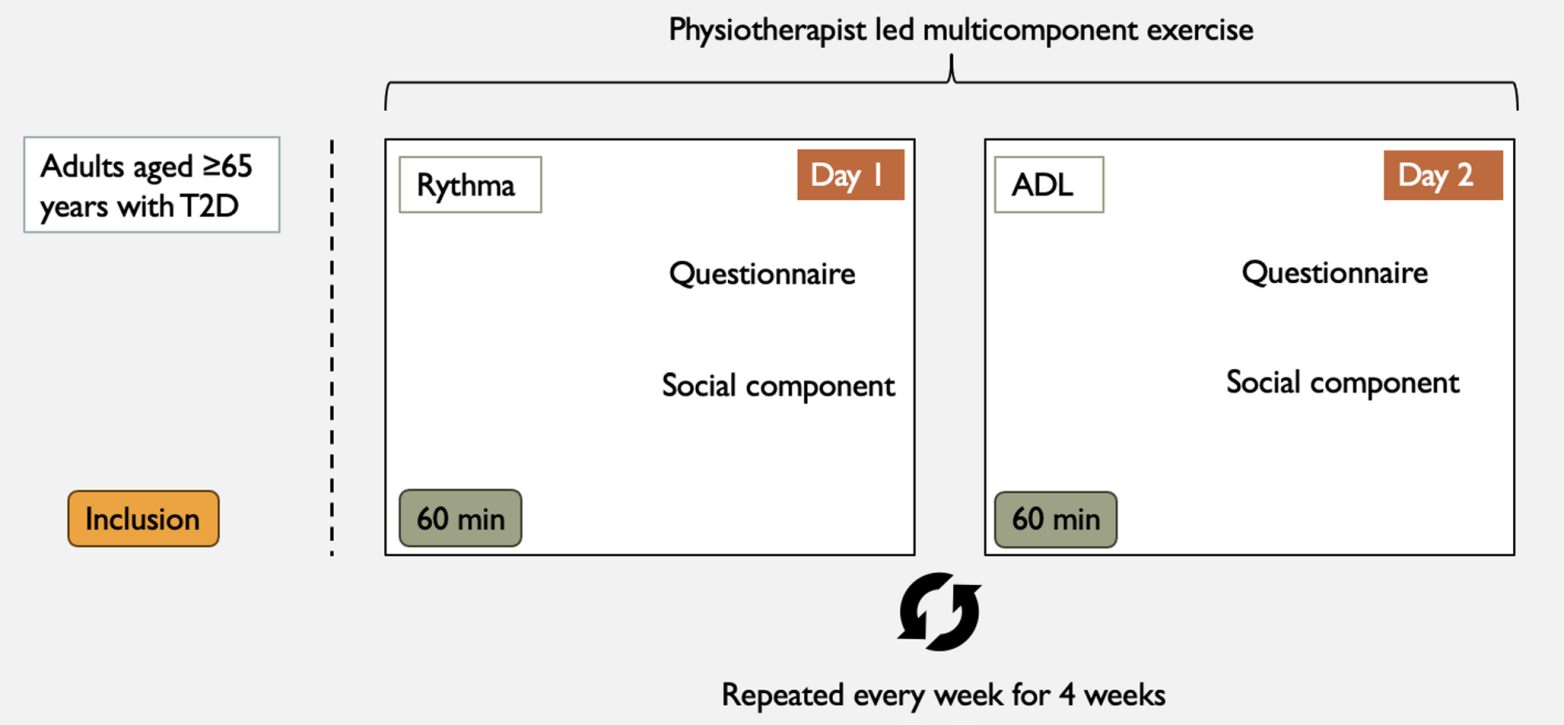
Feasibility set-up. Feasibility study illustrating the weekly multicomponent exercise elements after participant inclusion. Abbreviations: T2D = Type 2 diabetes, Rythma = Rhythm-based exercise and ADL = Activities of daily living

The intervention, termed RYMA (Rhythm-based and ADL Motor Activities), consisted of two components: activities of daily living (ADL) training and rhythm-based exercise (Rythma). The intervention period spanned four weeks following the screening and inclusion period. Participants attended two 60-minute sessions per week for four consecutive weeks, comprising one Rythma and one ADL-focused session (Fig. 1). Exercises were progressively intensified each week and designed to challenge both physical and cognitive functions (see Supplementary Material for the detailed exercise protocol). Each session concluded with a brief period of social interaction, supported by a café voucher that could be used for beverages and light snacks such as coffee, tea, fruit, or nuts. The duration of this period was not fixed but was self-determined by the participants. No research team member accompanied these interactions; however, the physiotherapists encouraged participation and guided the participants to the designated area. This component was included to encourage informal conversation, strengthen group cohesion, and enhance engagement and motivation to attend subsequent sessions.

After each session, participants completed a short questionnaire evaluating their experience of the training, perceived exertion, and overall enjoyment (see Appendix).

### Source of data

The feasibility and acceptability pilot study was conducted in accordance with the International Council for Harmonisation Good Clinical Practice (ICH-GCP) guidelines [25], all applicable regulatory requirements, and the Declaration of Helsinki for biomedical research involving human participants [26]. The trial was registered at ClinicalTrials.gov (NCT06745544) [27], reported to the North Denmark Region Committee on Health Research Ethics (protocol no. N-20240025), and to the North Jutland Research Department (ID no. F2024-197). All data were collected and securely stored using the Research Electronic Data Capture (REDCap) system v.13.1.37 [28]. Data analyses were performed using RStudio (version 2025.09.0 + 387; RStudio, PBC, Boston, MA, USA) and Microsoft Excel (version 16.101.2; Microsoft Corp., Redmond, WA, USA).

### Participants

The study aimed to recruit up to 12 participants with T2D, equally distributed by sex and of comparable age. Before the intervention, participants underwent fall risk assessment in accordance with the World Falls Guideline algorithm [29]. In addition, semi-structured interviews were conducted to collect demographic and health-related information, and relevant patient records were accessed with informed consent. All participants were informed that they were not eligible for inclusion in the subsequent randomized controlled trial, due to their prior knowledge of the intervention gained through participation in this study. Final inclusion was determined following screening according to the study’s eligibility criteria.

#### Inclusion and exclusion criteria

Eligible participants were men and women aged 65 years or older with a diagnosis of T2D for at least one year, living independently in their own homes, and able to provide written informed consent. People were excluded if they had prior experience with rhythm-based multitask exercise; were classified as being at high risk of falls (red category according to the World Falls Guidelines); had significant neurological (e.g., Parkinson’s disease, multiple sclerosis) or vestibular disorders, or had recently undergone major orthopedic surgery such as hip or knee replacement that could impair participation. Additional exclusion criteria were severe cognitive impairment (score < 8 on the Short Orientation–Memory–Concentration Test), complete dependence on walking aids, terminal illness, active cancer treatment, or inability to understand spoken or written Danish.

### Questionnaire

To ensure adherence and continuously monitor fall risk, a questionnaire containing 16 combined open- and closed-ended questions was administered. The questionnaire included items designed to assess acceptability, safety, tolerability, and adherence. After each training session, participants received a Danish version of the questionnaire (see Appendix) via e-mail [30]. To ensure compliance, physiotherapists were instructed to remind participants to complete the questionnaire. The same questionnaire was administered repeatedly after all eight sessions to follow response progression. In cases where participants experienced difficulty understanding specific questions, physiotherapists provided assistance accordingly. If a participant was unable to complete the survey electronically, a hard copy was provided and subsequently entered into REDCap (v. 13.1.37 - © 2025 Vanderbilt University) by a member of the research team.

### Statistics

#### Quantitative data

Descriptive statistics were reported as mean ± standard deviation (SD) or median with interquartile range (IQR), depending on data distribution. Categorical variables were summarized as count with percentages and compared using Fisher’s exact test. Normally distributed continuous variables were analysed with t-test, while non-normally distributed continuous variables were analysed with Wilcoxon rank-sum test.

#### Qualitative data

The qualitative text responses were analysed using Latent Dirichlet Allocation (LDA) [31] topic modelling. This approach was chosen because it is data-driven and inductive, allowing themes to emerge directly from the participants’ written responses rather than applying a predefined psychological or sociological framework. LDA is a probabilistic machine learning method that identifies latent structures in text by modelling documents as mixtures of topics and topics as distributions over words. In this study, the method was used to extract clusters of words that formed coherent themes.

The identified themes were derived empirically from word co-occurrence patterns in the data. Once topics were generated, the interpretation process was guided by principles of thematic analysis, in which the authors assigned conceptual meaning to clusters of words and made sense of them in relation to the research context [32].

The theoretical foundation of the analysis was therefore twofold. On the one hand, it relied on probabilistic topic modelling theory, as LDA provides a generative statistical model for discovering latent topics in text. On the other hand, it drew on the logic of thematic analysis and grounded theory, emphasizing inductive coding and the interpretive work of making sense of the patterns produced. All identified themes and groupings were subsequently reviewed and validated through manual examination to ensure conceptual clarity and contextual relevance. In this way, the analysis was driven by topic modelling while the interpretation was informed by an inductive thematic framework, allowing for a systematic yet flexible understanding of participants’ experiences.

## Results

### Feasibility

Eight participants with T2D were enrolled, evenly distributed by sex. Their baseline characteristics are presented in Table 1.

**Table 1:** Demographic

Variable	Male (*n* = 4)	Female (*n* = 4)	*P*-value
Nervous system, n (%)	0 (0%)	1 (25%)	1.00
Cardiovascular system, n (%)	2 (50%)	4 (100%)	0.43
Pulmonary system, n (%)	1 (25%)	0 (0%)	1.00
Gastro-intestinal system, n (%)	1 (25%)	2 (50%)	1.00
Urogenital system, n (%)	1 (25%)	0 (0%)	1.00
Gynaecological, n (%)	0 (0%)	1 (25%)	1.00
Muscle and joint, n (%)	2 (50%)	3 (75%)	1.00
Lymphatic system, n (%)	1 (25%)	0 (0%)	1.00
Skeletal, n (%)	0 (0%)	0 (0%)	-
Mucous membranes, n (%)	0 (0%)	2 (50%)	0.43
Age at screening, SD/IQR	71.75 ± 6.08	73.00 [72.75, 75.00]	0.66
Total score (Short orientation memory concentration test), SD/IQR	26.00 ± 2.83	27.00 [26.00, 28.00]	0.87
Clinical frailty score, IQR	1.00 [1.00, 1.50]	1.00 [1.00, 1.00]	0.45
Gait speed – Velocity, SD	1.41 ± 0.23	1.81 ± 1.11	0.53
Gait speed – Time, SD/IQR	2.89 ± 0.42	3.85 [3.35, 6.40]	0.25
TUG – Velocity, SD	8.32 ± 1.08	9.68 ± 1.42	0.18

Table 1 presents descriptive data of the study population, consisting entirely of participants with T2D, stratified by sex. Data are reported as percentages or as mean/median values, with variability expressed as either standard deviation (SD) or interquartile range (IQR), depending on the distribution.

Abbreviations: TUG = Timed-up-and-go test.

Attendance and adherence during the intervention period were high, with only a few days of absence and full completion of the study period by all participants (Table 2). Questionnaire completion was also consistently high across sessions, demonstrating good participant engagement.

**Table 2:** Attendance, injury and survey completion log

	Attendance		Injuries		Survey completion	
Session	Male	Female	Male	Female	Male	Female
1	3 (75%)	3 (75%)	0	0	4 (100%)	4 (100%)
2	3 (75%)	3 (75%)	0	0	2 (50%)	4 (100%)
3	2 (50%)	4 (100%)	0	0	4 (100%)	4 (100%)
4	3 (75%)	4 (100%)	0	0	4 (100%)	4 (100%)
5	4 (100%)	3 (75%)	0	0	4 (100%)	4 (100%)
6	3 (75%)	2 (50%)	0	0	4 (100%)	4 (100%)
7	4 (100%)	3 (75%)	0	0	4 (100%)	4 (100%)
8	3 (75%)	3 (75%)	0	0	4 (100%)	4 (100%)

Table 2 providing intervention log detailing participant attendance, survey completion, and any injuries sustained during training.

### Safety

No injuries or adverse events were recorded during any of the eight sessions. Self-reported discomfort, pain, or adverse sensations were observed in some participants, particularly during early sessions, but did not show an increasing trend over time. Perceived safety during exercise execution was initially moderate, with approximately half of participants reporting feeling safe in the first sessions. Over the course of the intervention, this proportion increased, indicating a growing sense of confidence and security among participants.

### Acceptability

The questionnaire comprised a combination of closed- and open-ended questions (see Appendix), which were analysed using appropriate methods to enable interpretation.

Participant adherence to the intervention was high, and satisfaction ratings with the training sessions were consistently favourable. The majority of participants reported the sessions as “satisfactory” or “very satisfactory”, with a gradual increase in “very satisfactory” ratings observed in later sessions. This trend suggests a positive trajectory in participant experience as familiarity and confidence developed.

Regarding the perceived level of challenge, participants initially rated the sessions as “not challenging” or “too easy.” As the intervention progressed, responses shifted toward “moderately challenging” and “appropriately difficult,” reflecting a gradual progression in perceived training intensity. Similarly, ratings of exercise difficulty evolved from being “too easy” at baseline toward “appropriate” in later sessions, supporting the intended adaptation of participants to the training regimen.

**Fig. 2:**
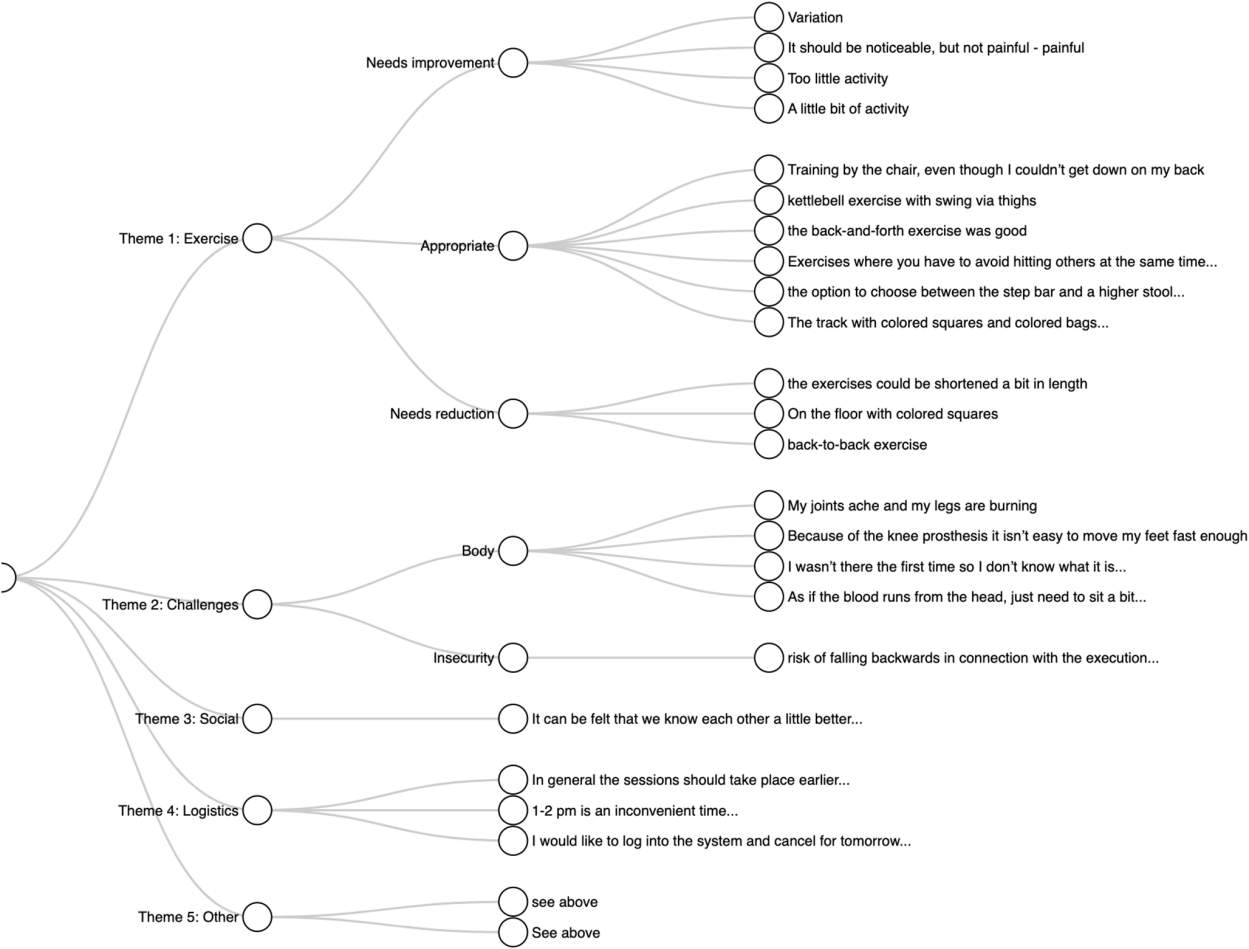
Coding three. Coding tree of all responses to open-ended questions, categorized into overarching themes. Themes and sub-themes are presented in English, while participant responses are translated to English from original Danish language response

The open-ended questions provided further insight into participants’ experiences. Thematic analysis (Fig. 2) identified five overarching themes: (1) Exercise, (2) Challenges, (3) Social, (4) Logistics, and (5) Other. Themes 1 and 2 (Exercise and Challenges) were further differentiated into subthemes, as illustrated in Fig. 2.

**Fig. 3:**
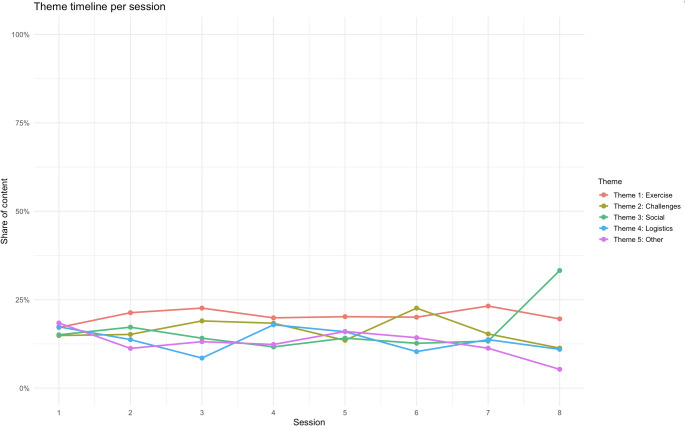
Progression in answers. Progression of thematic weight in participants’ responses over the course of the sessions

Progression across sessions revealed a shift in thematic emphasis. Early sessions were dominated by comments related to exercises (Theme 1). In the mid-sessions, participants more frequently highlighted bodily challenges and insecurities (Theme 2), while later sessions showed an increased focus on logistics and related aspects (Theme 4). Social aspects (Theme 3) and miscellaneous reflections (Theme 5) appeared throughout but declined toward the end as illustrated in Fig. 3.

## Discussion

### Feasibility, safety and acceptance

This feasibility study demonstrated that the DiaActive multicomponent exercise protocol is safe, well-accepted, and practically feasible for older adults with T2D. Participants completed the four-week intervention without adverse events, and adherence remained high. These findings align with previous studies showing that structured, supervised exercise protocol are both feasible and safe in this population [33–35].

Feasibility appeared to result from the program’s manageable structure—two weekly 60-minute sessions for four weeks—and its delivery by experienced physiotherapists in a familiar clinical setting. The limited duration, clear session organization, and small group format supported engagement and reduced logistical barriers. The program required minimal space, standard equipment, and modest staff time, suggesting that similar interventions are implementable in outpatient or community contexts.

Acceptability was closely linked to the program’s design. The combination of rhythm-based and ADL-focused exercises offered variety and relevance to everyday life, while the short social interaction at the end of sessions likely enhanced motivation and enjoyment. Together, these elements contributed to strong adherence and positive participant feedback, indicating both practical and experiential feasibility.

### The RYMA intervention

The RYMA intervention represents a novel approach that integrates rhythm-based dual-task training with functional ADL exercises and a structured social component. While these elements have each shown benefits in isolation, their combined use in a diabetes-specific fall-prevention context is new. The theoretical rationale lies in the potential synergy between motor, cognitive, and rhythmic engagement: rhythm provides an external timing cue that can enhance motor coordination and attentional focus, while ADL-based tasks promote relevance and transfer to everyday function. This integration may be particularly valuable for older adults with T2D, who often experience both physical and cognitive impairments that increase fall risk.

Our findings support the practical feasibility and acceptability of this integrated model. Participants responded positively to the varied and cognitively engaging format, reporting high satisfaction and growing confidence across sessions. These observations suggest that rhythm- and ADL-based dual-task training can be implemented safely and may foster adherence by making exercise both meaningful and enjoyable.

However, the clinical value of combining cognitive and motor elements remains hypothetical. While this feasibility study indicates promise, future randomized controlled trials are required to determine whether the integrated design produces measurable benefits in balance, cognition, and fall reduction compared with standard exercise protocol.

### Participant engagement, progression, and adherence

Participants reported increasing satisfaction as the training progressed, with early sessions perceived as easy and later sessions described as appropriately challenging - likely contributing to sustained motivation and attendance. Maintaining an optimal balance between challenge and perceived competence is known to enhance intrinsic motivation, which may explain the consistently high adherence and increasing satisfaction across sessions. In addition, this reflects that the exercise progression was well-calibrated and manageable, supporting both feasibility and participant tolerance. Importantly, the group-based format and interactive character of the sessions appeared to enhance motivation, enjoyment, and adherence—consistent with prior findings emphasizing the positive role of social and motivational factors in sustained exercise participation [36].

### Qualitative insights

The qualitative findings revealed a meaningful evolution in participants’ reflections throughout the program. Early responses centred on specific exercises, while later feedback highlighted bodily awareness, confidence, and social connectedness. This shift suggests a deepening engagement with the training process in which cognitive and physical engagement fostered self-efficacy and enjoyment. These insights underline that the social dimension was not merely supplementary but an essential driver of motivation and adherence—an aspect that will be explored systematically in the forthcoming RCT. Sex-specific themes were also observed, with men more often emphasizing strength and women highlighting balance—patterns consistent with earlier literature [37, 38]. The application of topic modelling provided a systematic framework for identifying these themes, and subsequent manual validation ensured reliable interpretation in accordance with recommendations for mixed-methods approaches [39].

### Next steps and perspectives

The present study provides evidence that the DiaActive multicomponent exercise protocol is feasible, safe, and acceptable for older adults with T2D. Based on these findings, the next logical step is to evaluate its effectiveness in a fully powered randomized controlled trial.

The planned trial will incorporate several refinements informed by the current feasibility outcomes. The intervention period will be extended to improve the potential for functional adaptation and to assess long-term adherence. Objective assessments—such as accelerometer-based activity monitoring, balance testing, and standardized functional performance measures—will be added to complement self-reported data. Broader inclusion criteria will allow participation of people with varying levels of frailty and comorbidity, improving generalizability. Moreover, evaluation of the program’s social and psychological effects will be integrated, reflecting the positive role of group-based and socially engaging elements observed in this feasibility study.

In addition to assessing clinical outcomes such as physical performance and fall risk, the forthcoming trial will explore potential cost-effectiveness and health-economic implications of implementing preventive exercise protocol for high-risk populations. While early evidence indicates that fall-prevention interventions can reduce healthcare costs [16, 40], such outcomes will be considered secondary to the primary evaluation of intervention efficacy. Overall, the present feasibility results provide a robust foundation for designing the definitive randomized controlled trial.

### Strength and limitations

This study has several important strengths. It is the first feasibility study of a rhythm-based, cognitive–motor intervention combined with physical exercise structured around activities of daily living in older adults with T2D—a high-risk but understudied group. Adherence was high, no injuries occurred, and participant satisfaction increased over time, underscoring both safety and acceptability. The inclusion of a structured social component after each session likely enhanced motivation, cohesion, and overall acceptability, highlighting the importance of integrating social engagement into exercise protocol for older adults. The mixed-methods design, combining quantitative measures with qualitative insights through LDA and thematic analysis, enabled a nuanced evaluation of feasibility. Conducting the program in a clinical setting with trained physiotherapists enhances external validity, while the group-based format further supported adherence and engagement.

However, the study also has limitations. The small sample size (*n* = 8) and short intervention period (four weeks) were suitable for a feasibility design but limit generalizability. Participants may have been more motivated than the broader population of older adults with T2D, introducing potential selection bias. Outcomes relied primarily on self-report, which is prone to bias, and the absence of a control group prevents causal inference.

## Conclusion

This feasibility study demonstrates that fall-preventive exercise integrating rhythm-based, ADL-oriented, and social components is safe, well-tolerated, and engaging for older adults with T2D. Adherence was high, no injuries occurred, and participant satisfaction increased across sessions, indicating strong feasibility and acceptability across all domains. Qualitative analyses revealed a shift from concrete comments on exercises in the early sessions toward reflections on bodily sensation, in later sessions. Collectively, these findings provide a strong foundation for implementation in a subsequent randomized controlled trial to evaluate the clinical and health-economic effects of the DiaActive program and its potential to improve functional capacity and reduce falls in older adults with T2D.

## Appendix


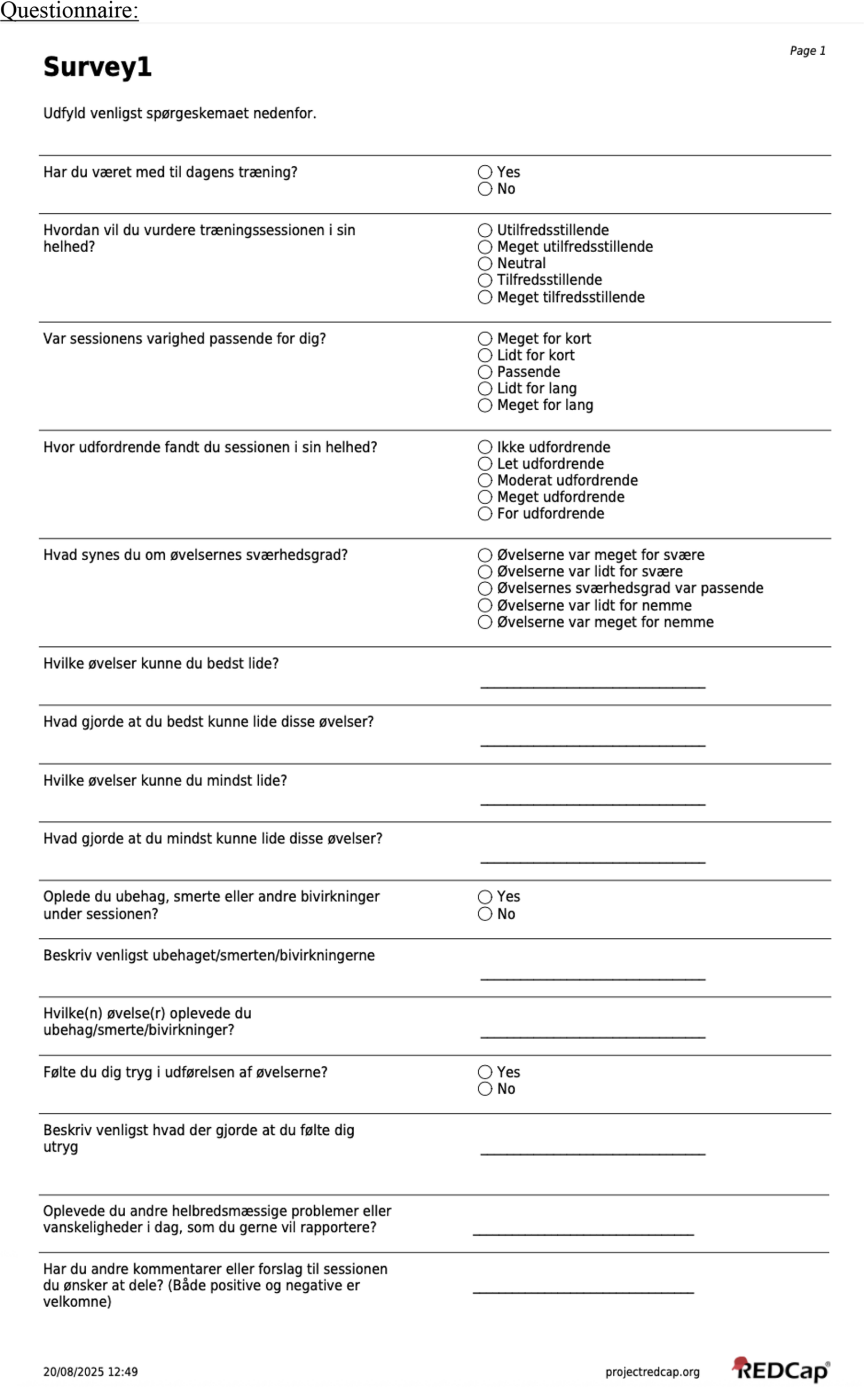



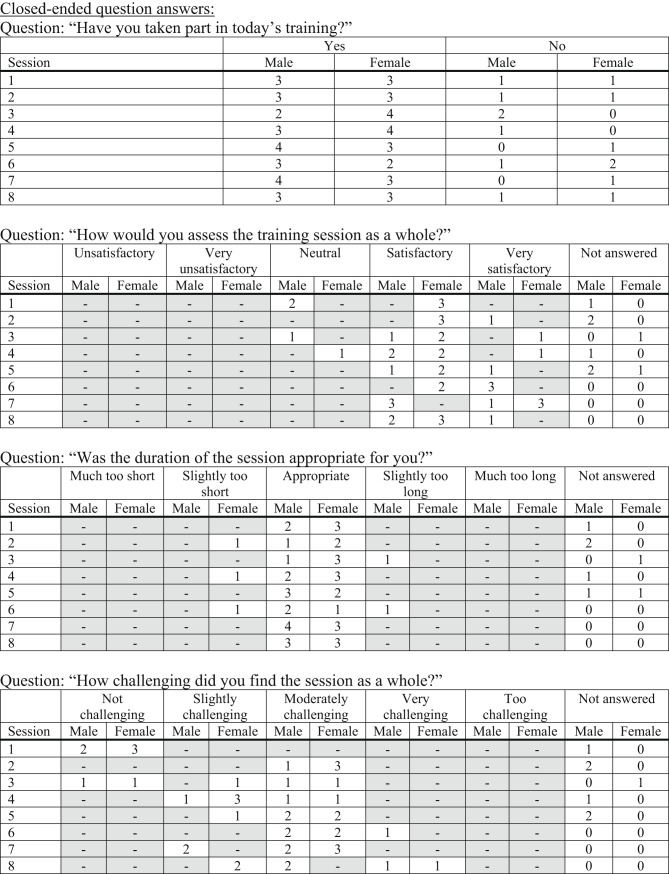



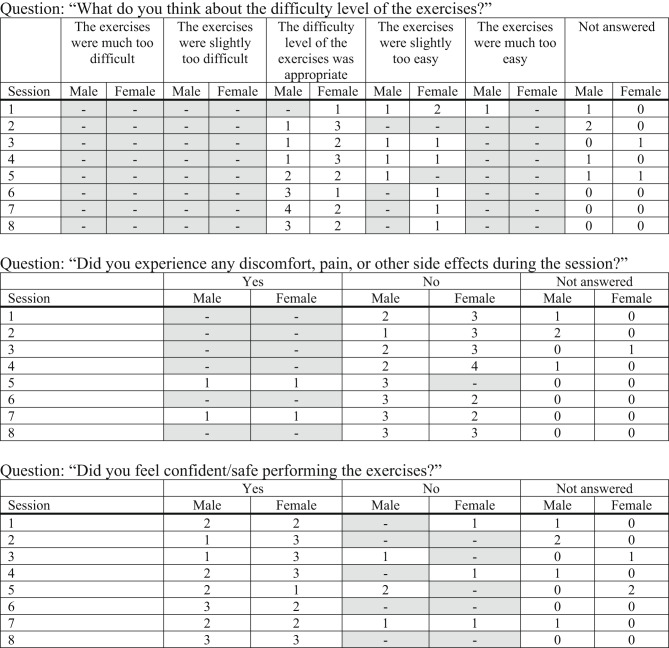


## Supplementary Information

Below is the link to the electronic supplementary material.

Supplementary Material 1

## Data Availability

The data are not available for sharing, as they contain personally identifiable information. Only the anonymized and published data in this study are available for sharing.

## Abbreviations

ADL: Activities of daily living
ICH-GCP: International Council for Harmonisation Good Clinical Practice
IQR: Interquartile range
LDA: Latent Dirichlet Allocation
REDCap: Research Electronic Data Capture
RYMA: Rhythm-based and ADL Motor Activities
Rythma: Rhythm-based exercise
SD: Standard deviation
SDCN: Steno Diabetes Center North Denmark
T1D: Type 1 diabetes
T2D: Type 2 diabetes

## Declarations

### **Use of artificial intelligence or large language models**

ChatGPT was used for optimizing the written academic English and grammar.
