# Supplementary material for: The DiaActive study: feasibility, safety and acceptability of a fall-preventive rhythm- and ADL-based exercise protocol for older adults with type 2 diabetes
Source: Aging Clin Exp Res. 2026 Feb 4;38(1):77. doi: 10.1007/s40520-026-03328-0 (PMC12901192; doi:10.1007/s40520-026-03328-0)
Supplement: Supplementary file 1 — Supplementary Material 1 [file 40520_2026_3328_MOESM1_ESM.docx]

# Training 1: Rhythm

Warm-up (10 min)

1b. Participants stand in a circle while music plays. They warm up the body in stages: toes, feet, ankles, shins, knees, thighs, the whole leg. Stomach/back with rotations. Fingers, hands, wrists, the whole arm. Neck.

1g. “Name and movement”: One participant says their name and performs a simple movement. The others repeat the name and the movement together. The next participant continues. Activates both body and cognition.

1c. Participants must stand up from the chair when they hear one specific sound, and sit back down at the sound of another.

Exercises focusing on music and movement (10 min)

2a. Participants walk slowly among each other to calm, quiet music. They focus on looking up and orienting themselves while walking. They must not bump into each other.

2c. Participants walk faster among each other to upbeat, cheerful music with a higher tempo. They focus on looking up and orienting themselves while walking. They also focus on ensuring that gaps are filled—when the music stops, they point to a gap that still needs to be filled. Repeat the exercise. They must not bump into each other.

2e. Participants continue walking among each other to upbeat music. Balls are introduced. Each time a participant with a ball passes another participant, the ball is handed over. Optionally, when the ball should be passed can be controlled by the instructor’s commands (e.g., piano tones).

Exercises with a social focus (10 min)

3d. Participants stand in a circle and receive a ball. Music plays. The ball is passed along in time with the music. When the instructor plays a specific tone, the ball must change direction. You can add that when the music stops, the ball also stops. Additional balls can be added to the circle that need to be kept track of.

Add one more ball.

Add forward or backward direction changes.

Exercises focusing on reaction (10 min)

4a. Freeze dance. Participants walk around the room and stop when the music stops. When the music stops, find a partner and stand back-to-back or make a “bridge” (hand to hand).

4b. A number of chairs are placed around the room. At a certain signal participants must quickly find a new chair and sit down—a mild form of musical chairs. Find the chair that is farthest from you.

Cool-down (10 min)

On chairs: Gentle stretches to calm music—for example in a circle where participants follow the instructor’s movements.

Training 2: ADL (Activities of Daily Living)

### Exercise | Description | Time

Introduction and warm-up | Mixed movements and stretches for arms, legs and body | 10 min

Station 1 (strength): Squats | Kettlebell squats (3 sets, 6–12 reps at ~70% 1RM) | 6 min

Rotation | — | 2 min

Station 2 (reaction): Reactive Tapping | The participant stands behind a plate. When a screen lights up, the participant steps onto the corresponding side of the plate. Participants take turns performing the exercise. | 6 min

Rotation | — | 2 min

Station 3 (strength): Stiff-Legged Deadlift | Stiff-legged deadlifts (3 sets, 6–12 reps at ~70% 1RM*) | 6 min

Rotation | — | 2 min

Station 4 (balance): Multitarget Stepping Task | Participant 1 chooses a color that Participant 2 must step on to complete the mat. After completion they switch roles. | 6 min

Rotation | — | 2 min

Station 5 (strength): Calf Raises | Calf raises (3 sets, 6–12 reps at ~70% 1RM*) | 6 min

Rotation | — | 2 min

Exercise 6 (coordination): Beanbag toss-and-catch | Participants stand facing each other and throw a beanbag. The thrower can give various commands while throwing, e.g., “catch with the left hand.” | 6 min

Cool-down | Gentle stretches and movements | 5–10 min

Training 3: Rhythm

Warm-up (10 min)

1a. Participants sit on chairs in a circle while music plays. They warm up the body in stages: toes, feet, ankles, shins, knees, thighs, the whole leg. Stomach/back with rotations. Fingers, hands, wrists, the whole arm. Neck.

1c. Participants must stand up from the chair when they hear one specific sound, and sit back down at the sound of another.

1e. Participants stand in a circle and mirror the instructor’s movements, which slowly increase in tempo and complexity (e.g., arm circles, knee lifts, hip swings).

Exercises focusing on music and movement (10 min)

2e. Participants continue walking among each other to upbeat music. Balls are introduced. Each time a participant with a ball passes another participant, the ball is handed over. Optionally, when the ball should be passed can be controlled by the instructor’s commands (e.g., piano tones).

2f. Participants move like different “characters” to music—for example a heavy elephant, a light bird, a dancing clown, a galloping horse, a sideways-moving crab, or a fast race car—given by the instructor.

Exercises with a social focus (10 min)

3a. Participants walk around the room, and each time they pass another participant they stop and shake hands (or high-five, etc.). Then they turn around each other, back-to-back, at an appropriate tempo, and continue walking.

3b. Participants stand facing each other in pairs. When the instructor plays a high tone, they high-five with the right hand. When the instructor plays a low tone, they high-five with the left hand. When the instructor plays a middle tone, they high-five with both hands. Can also be done with right and left foot to make it harder and incorporate balance.

3e. In pairs: One person makes a movement, the other mirrors it. After half a minute they switch roles. After a few switches the pairs rotate. You can set a focus, e.g., upper or lower body. Then switch to having participants choose a movement and link them into a movement chain.

Exercises focusing on reaction (10 min)

4c. Participants stand spaced out. When the instructor says a word (e.g., “jump”), they must do something else (e.g., sit down or turn around). The instructor changes words and movements continuously. Trains impulse control and reaction.

4d. The instructor plays a tone a varying number of times (1, 2, 3), and participants must react with the same number of steps, alternating backward and forward. Can be varied with other movements (sit and stand, etc.).

Cool-down (10 min)

On chairs: Gentle stretches to calm music—for example in a circle where participants follow the instructor’s movements.

Training 4: ADL

### Exercise | Description | Time

Introduction and warm-up | Mixed movements and stretches for arms, legs and body | 10 min

Station 1 (strength): Lunges | Lunges (3 sets, 6–12 reps at ~70% 1RM) | 6 min

Rotation | — | 2 min

Station 2 (reaction): Slalom with ball | The participant walks slalom between cones while dribbling a ball. | 6 min

Rotation | — | 2 min

Station 3 (strength): Kettlebell Swings | Kettlebell swing (3 sets, 6–12 reps at ~70% 1RM*) | 6 min

Rotation | — | 2 min

Station 4 (balance): “Tag” with an exercise | Participants face each other inside a square marked with cones. They perform 6 high knee lifts and try to tag each other. | 6 min

Rotation | — | 2 min

Station 5 (strength): Soleus raises | Soleus raises (3 sets, 6–12 reps at ~70% 1RM*) | 6 min

Rotation | — | 2 min

Exercise 6 (coordination): Goalkeeper drill | Participants face each other. One participant tries to shoot on goal while the other tries to save the ball. | 6 min

Cool-down | Gentle stretches and movements | 5–10 min

Training 5: Rhythm

Warm-up (10 min)

Same warm-up, but instead of standing still the participants walk in a large circle while swinging their arms and performing various warm-up movements. A high tone can be played to change direction in the circle; a low tone is a “trick” to stay in the same direction.

1d. Participants must stand up from the chair when they hear one specific sound, and sit down—on the neighbor’s chair—at the sound of another. That is, after standing up everyone moves one chair to the right.

1h. “Play-and-move”: The instructor plays a tone (or says a number), where each number corresponds to a movement (e.g., high = clap overhead, low = bend the knees). Participants must remember and perform the correct movement.

Exercises focusing on music and movement (10 min)

Bring Sally Up: Participants stand at their chairs and do sit-to-stand to the song. Stand when “up” is said and sit when “down” is said (the song is “Flower” by Moby—stop the song at 2:00).

Ghostbusters: Participants walk in a random pattern while the song plays. Pass the ball or give a high-five each time “Ghostbusters” is said. When a tone is played, walk backward; when it’s played again, walk forward, etc.

Exercises with a social focus (10 min)

Obstacle course: Slalom between cones. Balance on exercise mats. Over step benches and kettlebells. Sideways walking over small ropes.

Bomb game with one or two balls. We play music and the ball must be passed continuously; when the music stops, the “bomb” explodes (e.g., the participant does 2 squats or another task).

Mirroring chain: In pairs, participants must remember their own movement and their partner’s movement, and repeat the movements alternately with a new movement added at the end. (Similar to 3e: In pairs, one moves and the other mirrors. After half a minute switch roles. After some time the pairs rotate.)

Exercises focusing on reaction (10 min)

Musical chairs, find the correct chair: Participants walk around to upbeat music; when the music stops, they sit on the nearest chair—this becomes chair no. 1. Music starts again and participants walk. When it stops, they sit on a different chair than last time—this becomes chair no. 2. Next time the music stops the instructor calls “find chair no. X.” You can add 4–5 chairs; it’s about remembering your chairs.

Find the correct color: Each person is surrounded by four beanbags/cone tops in different colors. The instructor says a color, and the participant must touch the beanbag in that color—if “red” is called, place a foot on the red beanbag. Progression: you must use right or left foot. You can also use tones instead of saying the colors.

Cool-down (10 min)

On chairs: Gentle stretches to calm music—for example in a circle where participants follow the instructor’s movements.

Training 6: ADL

### Exercise | Description | Time

Introduction and warm-up | Mixed movements and stretches for arms, legs and body | 10 min

Station 1 (strength): Down to and up from a floor mat (bridge or crunch) | Lie down on the floor and get up 5 times × 3. Then do strength exercises. | 6 min

Rotation | — | 2 min

Station 2 (reaction): Reactive Tapping | Challenge by tapping with the opposite leg. Also with eyes closed—your partner says “NOW” to open your eyes and react. Or stand a little away from the screen. | 6 min

Rotation | — | 2 min

Station 3 (strength): Step-ups on a step bench | Step up and down with the same leg 10 times, then the opposite leg 10 times. The participant should remember to use the same leg up and down. They can coordinate stepping up and down alternately with their partner. | 6 min

Rotation | — | 2 min

Station 4 (coordination): Collect and throw beanbags to score points | Mark 10–20–100 points on the floor; participants try to hit the targets with beanbags. Use Post-it notes or painter’s tape to mark. | 6 min

Rotation | — | 2 min

Station 5 (strength): Calf Raises | Calf raises (3 sets, 6–12 reps at ~70% 1RM*) | 6 min

Rotation | — | 2 min

Exercise 6 (balance and reaction): Perturbation exercises | Participants attempt to clap their hands into the opponent’s hands while simultaneously trying to avoid the opponent clapping their hands, by pulling their own hands away. | 6 min

Cool-down | Gentle stretches and movements | 5–10 min

Training 7: Rhythm

Warm-up (10 min)

1f. “Movement wave”: Participants stand in a circle. One participant starts a movement (e.g., arm up) which is sent around like a wave. After one round the direction changes.

Safari relay: In pairs, one at a time runs to a cone. Move like a tall giraffe, heavy elephant, sideways crab, fast gazelle, high knee lifts like a flamingo. (Let participants draw the animal on a slip of paper.)

Exercises focusing on music and movement (10 min)

2e. Participants continue walking among each other to upbeat music. Passing the ball is controlled by the instructor’s commands (e.g., piano tones). At some point the walking changes to backward and sideways. Optionally, balance a beanbag on the head.

Ghostbusters: Participants march on the spot in time with the music. Each time “Ghostbusters” is said, pass the ball. Along the way the walking movement changes to high knees, heel kicks, side-to-side (dance). A tone indicates that you should turn around in place.

Exercises with a social focus (10 min)

Two-by-two dance competition: You must move to the rhythm of the music and numbers are called during the song. 1 = hand to hand (bridge), 2 = back to back, 3 = side by side/normal walk, 4 = walk backward side by side.

Tail tag: A tag game in chaos, where everyone has a “tail” and tries to steal each other’s tails.

Exercises focusing on reaction (10 min)

Freeze dance with colors: when the music stops, a color is called and you must find and go to it.

Follow-the-leader (movement): They work in pairs. One leads and the other follows; you can only move forward/back or sideways. When a tone is played, switch so the other person leads.

Cool-down (10 min)

On chairs: Gentle stretches to calm music—for example in a circle where participants follow the instructor’s movements.

Training 8: ADL

### Exercise | Description | Time

Introduction and warm-up | Mixed movements and stretches for arms, legs and body | 10 min

Station 1 (strength): Squat | Squat (3 sets, 6–12 reps at ~70% 1RM). To ensure sufficient depth, place a kettlebell on the floor for them to pick up. | 6 min

Rotation | — | 2 min

Station 2 (reaction): Slalom with ball | The participant walks slalom between cones while dribbling a ball. | 6 min

Rotation | — | 2 min

Station 3 (strength): Carrying with kettlebells | Jog, sideways walking, and possibly backward (3 laps around the cones). | 6 min

Rotation | — | 2 min

Station 4 (balance): Multitarget Stepping Task | Participant 1 chooses a color that Participant 2 must step on to complete the mat. After completion they switch roles. (Vary by changing color mid-course, timing it, balancing a beanbag on the head—beanbags are placed on the mat and you must step on one color while collecting beanbags of another color.) | 6 min

Rotation | — | 2 min

Station 5 (strength): Soleus raises | Soleus raises (3 sets, 6–12 reps at ~70% 1RM*). | 6 min

Rotation | — | 2 min

Exercise 6 (coordination): Goalkeeper drill | Participants face each other. One participant tries to shoot on goal while the other tries to save the ball. | 6 min

Cool-down | Gentle stretches and movements | 5–10 min

Multitarget Stepping Task (notes)

• Can be tried while dribbling a ball. Also sideways and backward—and timed. Note: stopwatch?

• Can also be tried by throwing beanbags and collecting a specific color. For example: step on red, but collect blue beanbags (this also trains the function of picking something up from the floor).

Abduction exercises
